# Editorial: Enzyme immobilization technologies and their biomanufacturing applications

**DOI:** 10.3389/fbioe.2023.1256181

**Published:** 2023-07-31

**Authors:** Jiandong Cui, Ismail Ocsoy, Mohamed Abdelraof Mahmoud, Yingjie Du

**Affiliations:** ^1^ State Key Laboratory of Food Nutrition and Safety, Laboratory of Industrial Fermentation Microbiology, Ministry of Education, Tianjin University of Science and Technology, Tianjin Economic and Technological Development Area (TEDA), Tianjin, China; ^2^ Department of Analytical Chemistry, Faculty of Pharmacy, Erciyes University, Kayseri, Türkiye; ^3^ Microbial Chemistry Department, Genetic Engineering and Biotechnology Research Division, National Research Centre, Cairo, Egypt

**Keywords:** biocatalysis, enzyme engineering, immobilization, biomanufacturing, industrial biotechnology

With the looming apprehension of climate change, extensive environmental deterioration and mass extinctions, the transition to a greener, environmentally friendly, and sustainable production of liquid fuels and platform chemicals has become imperative. Biotechnological processing by enzymes has been used widely in a wide range of industrial sectors including chemicals, pharmaceuticals, food and feed, detergents, pulp and paper, textiles, energy, materials, and polymers. From the last several years ligninolytic enzymes find applications in numerous industrial processes (Sheldon et al., 2020; Wu et al., 2021). However, their lower catalytic efficiencies and operational stabilities limit their practical and multipurpose applications in various sectors of the current industrial processes (Ren et al., 2019; Feng et al., 2022). It is necessary to focused primarily on recent trends in green enzyme evolution and immobilization biotechnology around the potential industrial applications of enzymes in various sectors of the modern industry.

To solve these problems, enzyme immobilization approaches have been adopted as parallel or mutually auxiliary strategies for improving performance of enzyme. Recent reports show efforts on improving both enzymatic activity and stability through immobilization (Bilal et al., 2023). The major issue for obtaining improved biocatalysts using in industrial biotechnology is how to remold enzyme with mild, simple, and effective methods, especially in the actual complex catalytic environment. With the rapid development of chemistry, computer, materials and other disciplines, more and more methods have been used to optimize the design of immobilized enzyme. Enzyme immobilization is growing rapidly and will become a powerful norm in bio-catalysis with much controlled features, such as selectivity, specificity, stability, resistivity, induce activity, reaction efficacy, muti-usability, improved mass transfer efficiency, high catalytic turnover, optimal yield, ease in recovery, and cost-effectiveness. In addition, enzyme immobilization strategies for complex enzyme processes such as multi-enzyme catalysis and non-aqueous enzyme catalysis should be proposed as soon as possible (Ren et al., 2019).

In total, four articles were published in this Research Topic summarizing various aspects of enzymes like immobilization, characterization, improving catalytic attributes and applications in multiple sectors. For example, Ma et al. reported immobilization and property of penicillin G acylase on amino functionalized magnetic Ni_0.3_Mg_0.4_Zn_0.3_Fe_2_O_4_ nanoparticles. The activity of the immobilized PGA reached a maximum of 7,121.00 U/g. Meanwhile, the immobilized PGA exhibited higher stability against changes in pH and temperature. Furthermore, the immobilized PGA revealed excellent cycling performance. He et al. explored the high level expression of nicotinamide nucleoside kinase from *Saccharomyces cerevisiae* in *Escherichia coli*, and developed one-step method to purify and immobilize nicotinamide nucleoside kinase. After determination and conversion, the enzyme activity in the fermentation broth reached 14.75 IU/mL, and the specific enzyme activity after purification was 2,252.59 IU/mg. Moreover, the activity of the immobilized enzyme remained above 80% after four cycles.

Organic–inorganic hybrid nanoflowers technology has been emerged as an effective immobilization method. This method has motivated a considerable interest in exploiting them as a potential matrix for biomolecule immobilization due to their simple synthesis, high efficiency, great promise of enhancing biomolecule stability, activity and even selectivity. Recent years, many efforts have focused on this topic to develop biomolecule-inorganic hybrid nanoflowers with potential applications (Cui and Jia, 2017). Demirbaş et al. (2023) developed a facile synthesis method for hybrid nanoflowers using glycine and phenylalanine (AA-hNFs). The AA-hNFs exhibited a uniform flower shape and peroxidase-like activities. Therefore, these amino acid-inorganic hybrid nanoflowers could be applied as industrial biocatalysts, biosensors, and bioanalytical devices. Recent years, molecular dynamics simulation method has been used to understand the structural and dynamic aspects of distinct enzyme immobilization strategies. Bhattacharjee et al. discussed how molecular dynamic simulations have been employed to characterize the surface phenomenon in the enzyme immobilization procedure. They summarized computational studies on the immobilization of enzymes using nanoparticles, self-assembled monolayers, graphene and carbon nanotubes, and other surfaces. Until now, there are the few recent proposals for predicting immobilization protocols using molecular dynamics simulation method. Altogether, all articles in this Research Topic evidently demonstrate the importance of immobilized enzymes in a variety of biomanufacturing applications.
